# Massive spinal epidural infantile hemangioma, image findings, and treatment: a case report and review of literature

**DOI:** 10.1007/s00256-024-04570-1

**Published:** 2024-03-21

**Authors:** Youssef Ghosn, Yara Jabbour, Farah Abou Zeid, Nawaf Jurdi, Riad Khouzami, Hicham Moukaddam

**Affiliations:** 1 Department of Diagnostic Radiology, American University of Beirut, Riad El-Solh, P.O. Box 11-0236, Beirut, 1107 2020 Lebanon; 2 Department of Pathology, American University of Beirut, Beirut, Lebanon

**Keywords:** Epidural Infantile hemangioma, Beta-blockers, Vascular tumor, Classification

## Abstract

Spinal involvement of infantile hemangiomas is rare with the predilection to involve the epidural space. A proper diagnosis might be challenging due to the atypical location and variable/inconsistent use of the International Society for the Study of Vascular Anomalies (ISSVA) classification by radiologists, pathologists, and clinicians. A proper diagnosis of epidural infantile hemangioma is key due to the different aggressiveness of the treatment options with inconstant literature regarding the best available treatment. Herein, we present a case of a massive epidural infantile hemangioma successfully treated with only beta-blocker. We discuss the clinical, MRI, CT, ultrasound, and histological features of this lesion as we review the literature with the objective of addressing some of the confusion surrounding the subject.

## Introduction

Infantile hemangiomas are hamartomatous lesions that result from proliferations of vascular endothelial cells [1]. They are estimated to affect approximately 4% of infants, most commonly found in the skin, and typically regress without surgical treatment [2, 3]. In contrast, the prevalence of neuraxial involvement by infantile hemangioma is low comprising around 1% of all hemangiomas. When present, neuraxial hemangiomas have the propensity to involve the extradural spine [4] and, in contrast to cutaneous hemangiomas, both surgical and medical management have been proposed in the literature. However, the classification and nomenclature used to describe endothelial malformations has been a source of confusion with variable adoption of the terminology suggested by the International Society for the Study of Vascular Anomalies (ISSVA). This is especially true when describing CNS vascular lesions in the pediatric population [5]. Moreover, sometimes it might be difficult to separate infantile hemangiomas from other vascular tumors and vascular malformations based on histology alone requiring more reliance on the clinical picture. This makes the literature about the subject somewhat confusing with possible misuse of the term hemangioma, making a proper evaluation of the prevalence, clinical course, image characteristic (including tumor location), histology, and treatment difficult [5–7]. Herein, we present a case of a massive infantile epidural hemangioma in a neonate, showing multilevel foraminal extensions and successfully treated with only beta-blockers as we discuss the clinical, MR, CT, US, and histological features of this lesion aiming to clear up some of the confusion about the subject.

## Case presentation

A 3-week-old female neonate presented to the neurosurgery team for evaluation for lower extremities weakness. On history taking, the patient was born at full term with a birth weight of 3.145 kg to a healthy mother. The mother had received Enoxaparin for antiphospholipid syndrome during her pregnancy with no other complications. The baby was born by cesarean section due to breach position. On physical examination, the patient was noticed to have a prolapsed anus, a midline lumbar dimple, negative deep tendon reflexes, presence of primitive reflexes, distal paresis of the right leg with flaccid deformity, proximal distal paresis of the left leg and laxity of the anal sphincter.

**Fig. 1:**
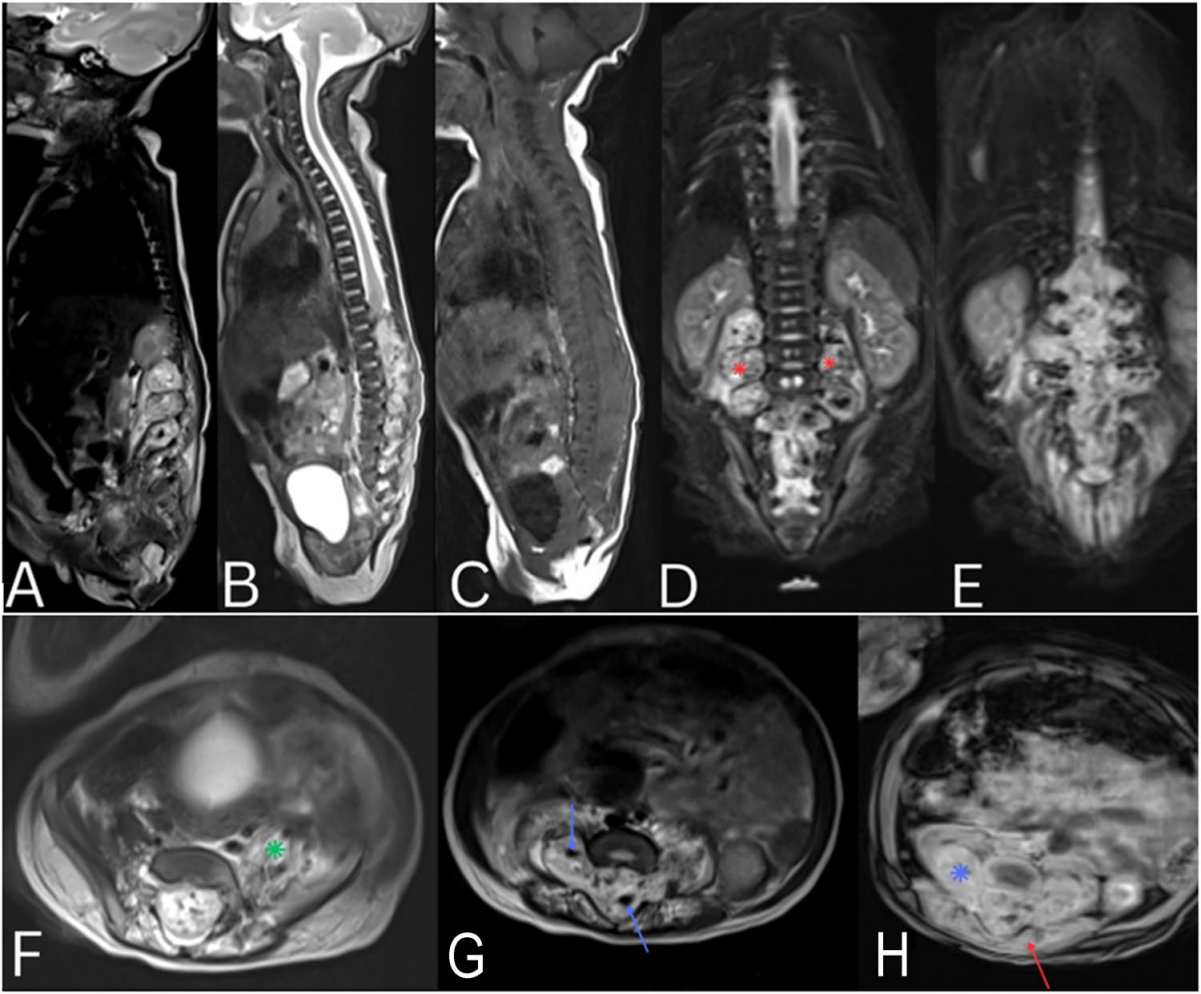
Sagittal T2 (images **A** and **B**), Sagittal T1 (image **C**), Coronal STIR (images **D** and **E**), axial T2 (images **F** and **G**) and axial T1 post gadolinium (image **H**) images of the spine showing a large infiltrative avidly enhancing (blue asterisk, image **H**) tumor occupying the spinal canal from T10 to S1 with a multilevel Dumble shaped extra spinal components protruding from multiple neural foramina (red asterisk image **D**). The mass showed high signal intensity on T2 weighted images (images **A**, **D**, **F**, **E**, **G**), low signal intensity on T1 weighted images (image **D**) and extensive flow voids (blue arrows, image **G**). Note is made of edema in the left psoas muscle (green asterisk, image **F**) and involvement of the adjacent paraspinal muscles (red arrow, image **H**). No signal abnormality was detected at the rest of the spinal cord

An MRI of the brain and spine was done (Fig. 1) revealing a large infiltrative avidly enhancing tumor occupying the spinal canal from T10 to S1 (8 vertebral levels) for a craniocaudal distance of 8.3 cm with extra spinal components infiltrating and protruding from multiple expanded neural foramina bilaterally (Dumble chapped) and abutting the psoas muscles with involvement of some adjacent paraspinal muscles. The mass showed high signal intensity on T2-weighted images, low signal intensity on T1-weighted images and extensive flow voids suggesting a vascular component. There was no evidence of microscopic or macroscopic fat on fat-suppression and in-phase and out-of-phase sequences. Increase in T2 signal was noted in the bilateral psoas muscles, more significantly on the left, which could be related to muscle edema secondary to denervation changes. The spinal cord was obliterated at the level of the lesion. No signal abnormality was detected at the rest of the spinal cord.

**Fig. 2:**
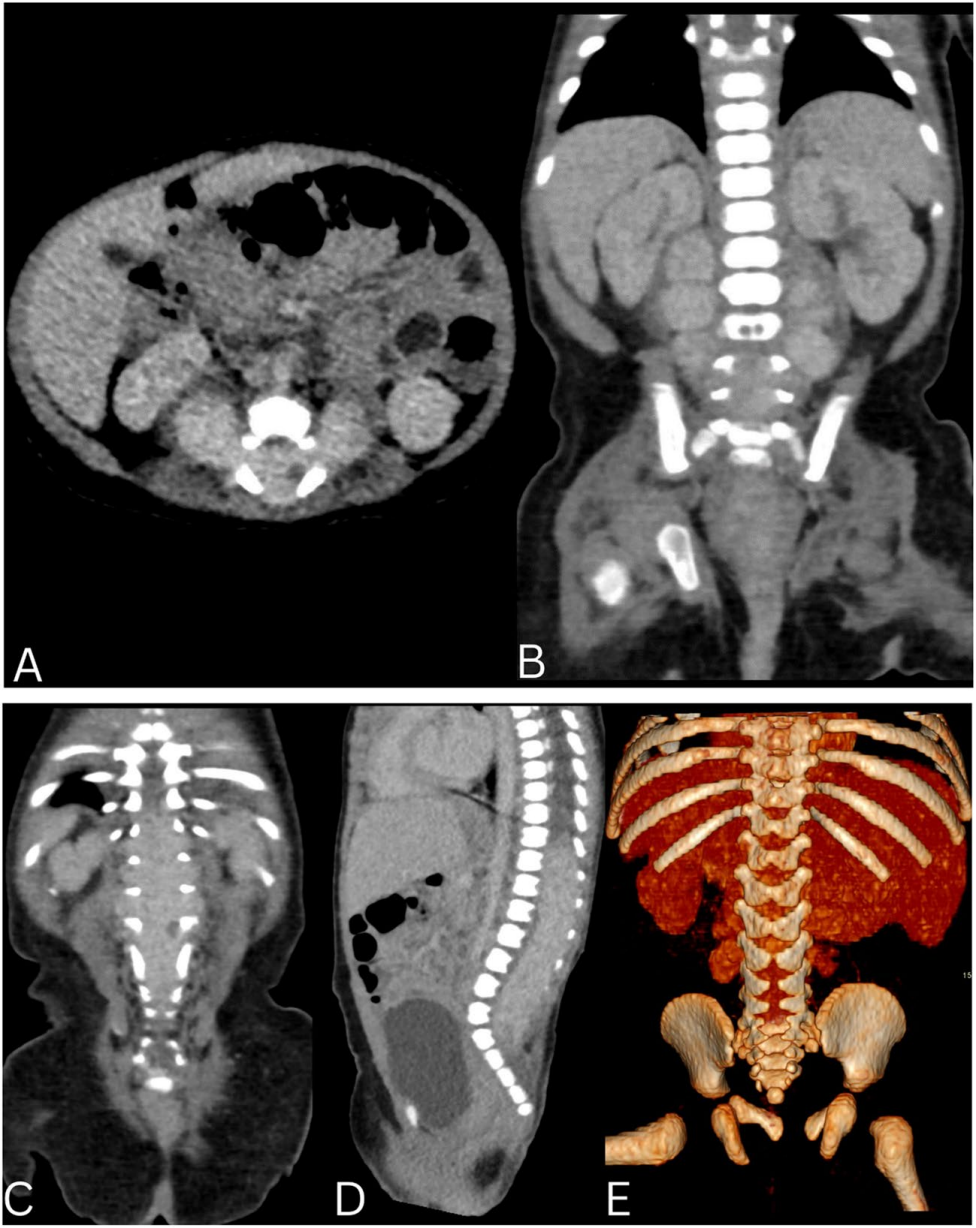
Axial (image **A**), Coronal (image **C** and **D**) contrast enhanced CT scan of the spine with 3D reconstruction (image **E**) showing a high attenuation mass occupying the lower thoracic and lumber spinal canal, extending through multiple neural foramina forming a Dumble shaped appearance (image **A**). 3D reconstruction images show multilevel posterior elements failing to fuse in keeping with Spina bifida (image **E**)

Contrast-enhanced CT images (Fig. 2) showed a high attenuation mass expanding the spinal canal and neural foramina. No calcifications were seen. Non-fusion of the posterior elements from L4 caudally was noted in keeping with spina bifida. The differential diagnosis included neuroblastoma, plexiform neurofibroma, schwannoma and hemangioma.

**Fig. 3:**
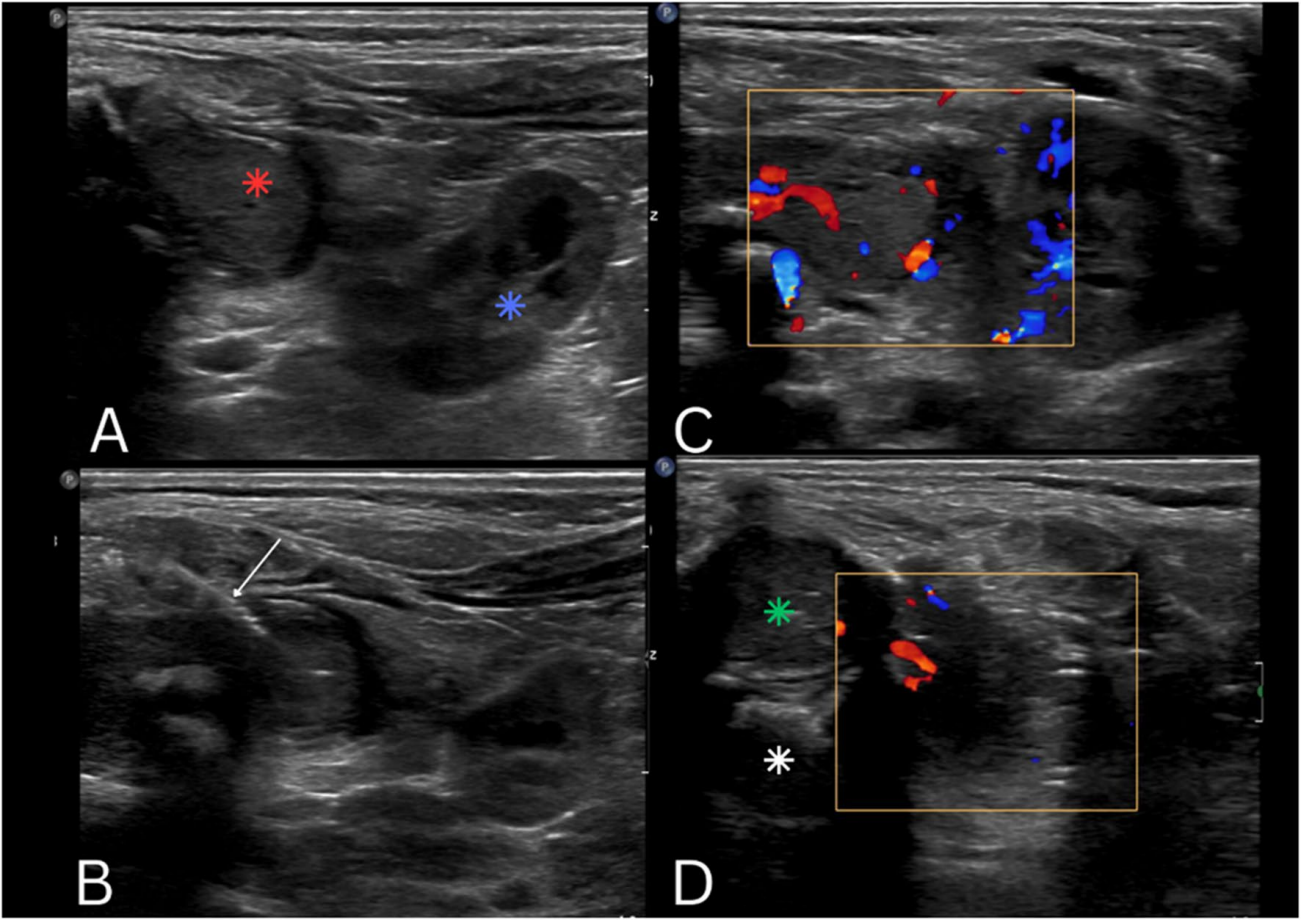
Transverse ultrasound images of the spine and right paraspinal region during transcutaneous biopsy. There is a hypoechoic structure occupying the pine canal (green asterisk, image **D**) with the vertebral body anteriority (white asterisk, image **D**) with a protruding component through the right neural foramina (red asterisk, image **A**) adjacent to the right kidney (blue asterisk, image **A**). The lesion shows increased flow on doppler images, comparable to that of the adjacent kidney (image **B**). Note is made of the biopsy needle (white arrow, image **B**)

An ultrasound-guided core biopsy (Fig. 3) of this mass was done showing hypoechoic protruding masses with high vascularity on Doppler images. The specimen was sent for histological analysis.

**Fig. 4:**
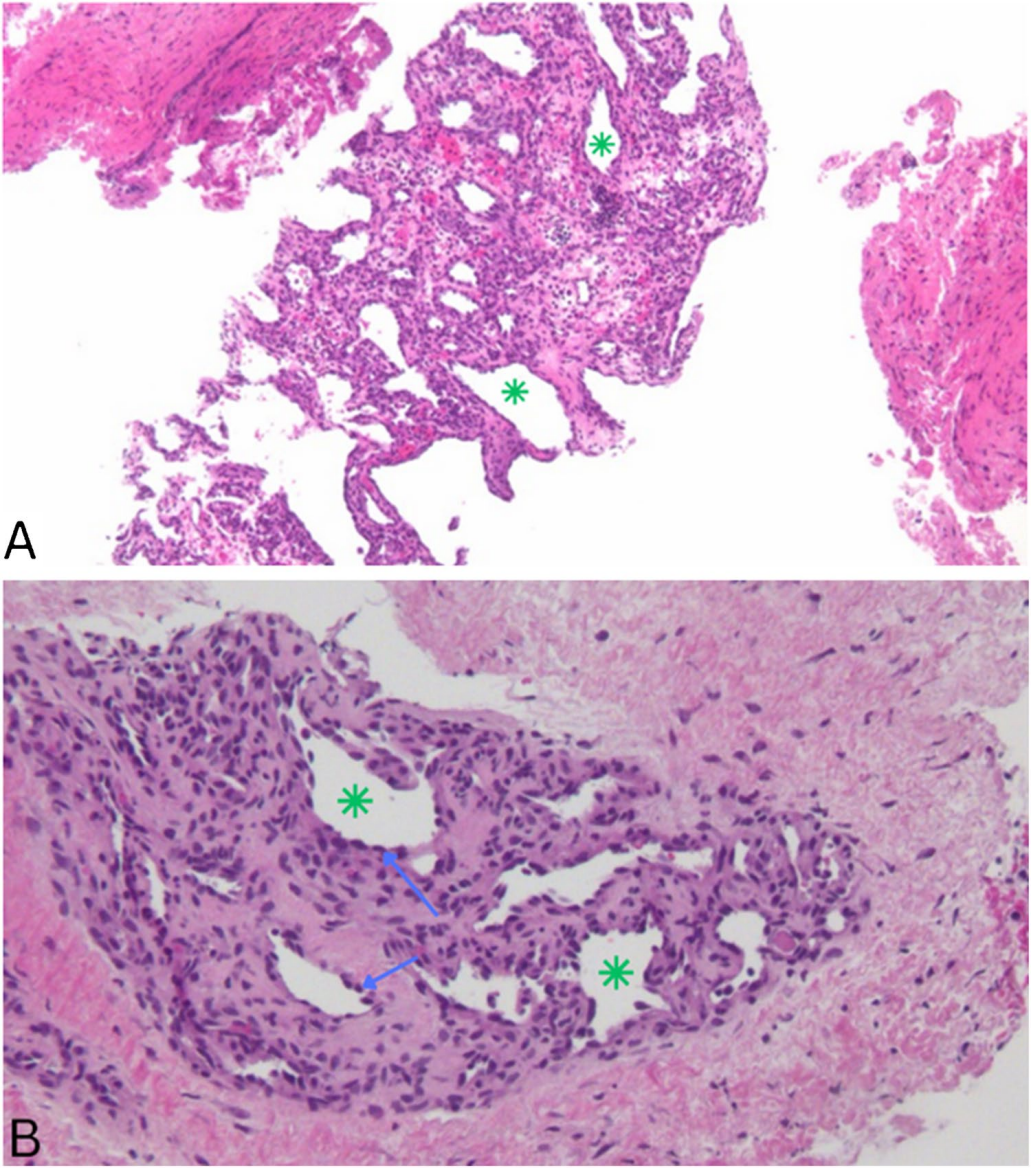
Histologic examination of the specimen showed lobular proliferation of capillary sized vascular spaces (green asterisks) that are lined by bland endothelial (blue arrows)

Histologic examination of the specimen (Fig. 4) showed lobular proliferation of capillary sized vascular spaces that are lined by bland endothelial cells with some areas of focal intravascular thrombosis. The findings were described as typical for capillary hemangioma.

The decision was made to not proceed with surgical excision at such a young age due to the high risk of complete paralysis. The patient was started on Propranolol 15 mg two times a day for 6 months, until the next radiologic examination.

**Fig. 5:**
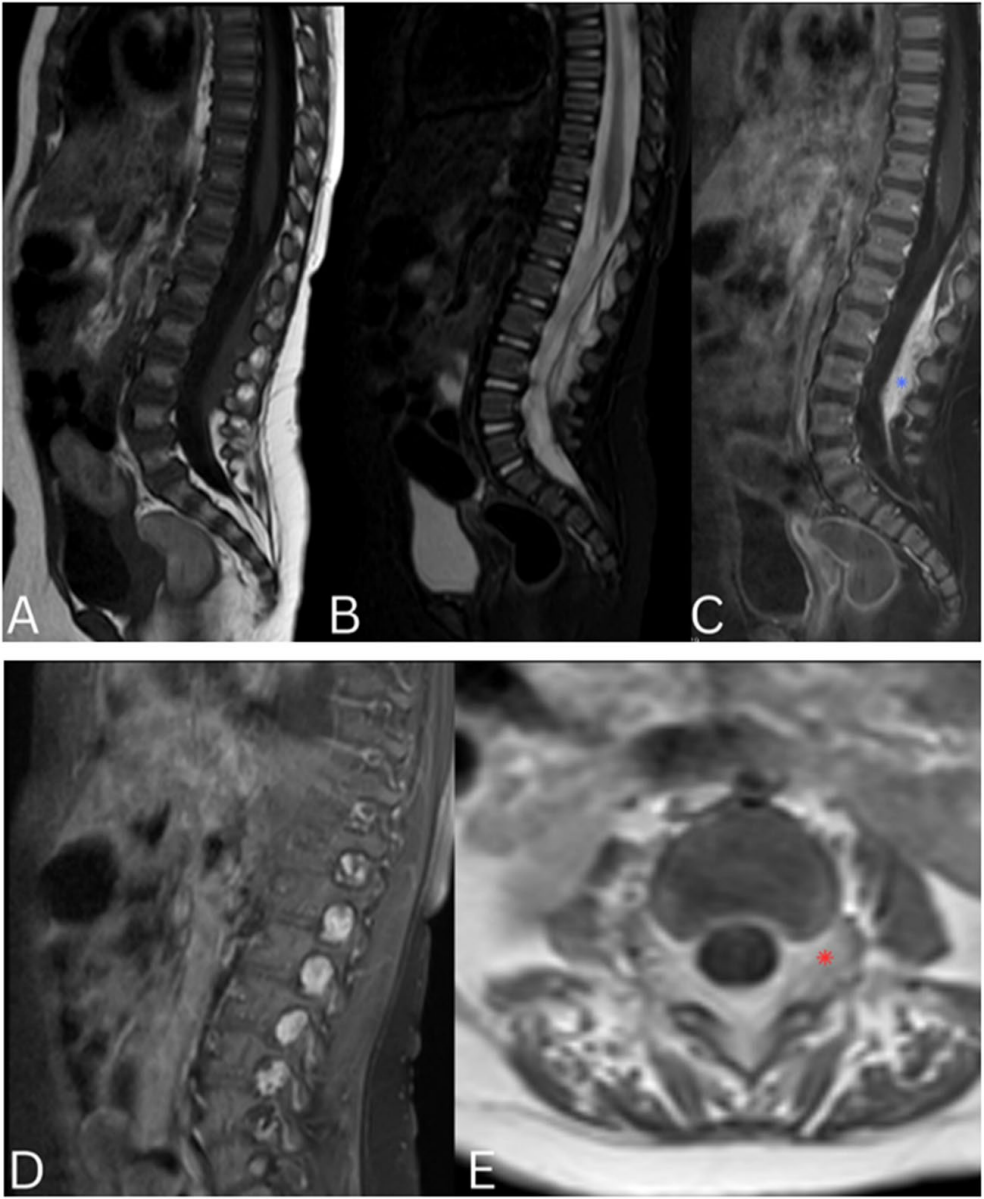
Sagittal T1 (image **A**), Sagittal T2 (image **B**), Sagittal T1 post gadolinium (image **C** and **D**), and axial T1 post gadolinium T2 (image **E**) images of the lumber spine 6 months after beta blocker therapy showing significant decrease in size and extent of the mass with residual posterior epidural component (blue asterisk, image **C**) with decrease but persistent areas of foraminal extension (red asterisk, image **E**). Not is made of significant decrease in the flow voids seen on prior MRI

Follow-up MRI in 6 months (Fig. 5) showed significant decrease in size of the mass, with residual posterior epidural component, significant decrease of the foraminal extension however still present from T12-L1 to L3-L4, and resolution of the cord compression. There was no myelomalacia. The mass still exhibits intense enhancement however there is significant decrease in the internal flow voids suggesting progressive involution (involution phase). The original images showing extensive vascularity suggest a proliferative phase. Clinically the patient showed improvement in the left lower extremity motor power.

The clinical behavior of the tumor, along with the imaging and histological features were consistent with infantile hemangioma.

## Discussion

The ISSVA classifies vascular anomalies into 2 major categories based on clinical and cellular behavior: 1. vascular tumors such as hemangiomas, which arise by endothelial hyperplasia. 2. vascular malformation, defined as a congenital or developmental morphogenic anomaly of various vessels with a normal endothelial turnover and normal growth rate [8]. Hemangiomas are divided into infantile or congenital hemangiomas with the previously known capillary, capillary/cavernous, and strawberry hemangiomas now categorized as “infantile hemangiomas” [4, 9, 10]. However the term capillary hemangioma seems, in some instances, to be used interchangeably by pathologists for infantile hemangioma and congenital hemangiomas [11].

Vascular malformations have been subdivided into two subgroups: slow- or low-flow malformations, and fast- or high-flow malformations. Low-flow include capillary, venous, lymphatic, and mixed malformations. The ISSVA classification has abandoned the terminology of cavernous hemangiomas (or cavernous malformations), instead the terminology of venous malformation is now used. However, it was not completely accepted by neurosurgeons; the term “cavernous malformation” is still used, and other terminologies that includes “cavernous” (e.g., cavernous hemangioma or cavernous angioma) are also used [7].

Regardless, infantile hemangioma might not be diagnosed by relying solely on pathology. The clinical history, classically presenting as a lesion appearing in the first few weeks of life, with the proliferative phase then reaching a plateau followed by a slow involuting phase, in addition to the imaging findings can be an integral elements of the diagnosis [12]. Moreover, immunohistochemistry and genetic studies may not be available for detailed pathological assessment. Therefore, confidently deciding to categorize a lesion based on histology alone might not be possible.

Epidural hemangiomas can present clinically with progressive myelopathy. The prevalence of epidural hemangiomas in the pediatric population is unknown with neuraxial involvement reported to compromise around 1% of all hemangiomas [4]. Of the 1454 patients listed with infantile hemangioma reviewed by Viswanathan V et al. (2009), 15 (1.0%) had involvement of the CNS, 6 (0.4%) of those were intraspinal, all of with epidural in location [4]. Cohorts by Drolet BA et al. (2010) showed a prevalence of 50% (21 cases) of “intraspinal” hemangiomas in patient with infantile hemangioma of the lumbosacral skin (*n* = 41) without specifying the specific spinal location (epidural, intradural or medullary) [13]. This study suggests that the prevalence of epidural hemangiomas in the pediatric population might be under-estimated, moreover not specifying the exact location of the hemangioma suggest difficulty in pinpointing the precise site relative to the spinal canal. This issue is especially relevant with large infiltrative masses. Schumacher WE et al. (2011) showed a prevalence of 45% (9 cases) of intraspinal hemangioma in patients with spinal dysraphism (*n* = 20). These were characterized as only extradural, present in six children, or only intradural, present in one patient with two children having both intra- and extradural hemangiomas [14].

### Imaging

Depending on the stage of their natural history, infantile hemangiomas have a characteristic appearance on cross-sectional imaging and angiography. During the proliferative phase, CT and MR imaging demonstrate a homogeneous soft-tissue mass, which shows intense enhancement. Proliferating hemangiomas are hypointense to isointense to muscle on T1-weighted images and hyperintense on T2-weighted images with associated flow voids [15]. Occasionally, internal signal heterogeneity may be seen, indicating thrombus or prior hemorrhage [16, 17]. Doppler sonography shows numerous intralesional and perilesional vessels with a high peak arterial Doppler shift [18, 19].

If preformed, angiography demonstrates enlarged arterial feeders, pooling of contrast within tumoral vascular spaces [20], possible early draining veins and occasional arteriovenous shunting. During the involuting phase, hemangiomas show less enhancement, show less fast-flow vascularity, and appear more heterogeneous. Ultimately, fibrofatty tissue characterizes the final involuted phase [4, 20].

Intracranial/intraspinal infantile hemangiomas and cutaneous hemangiomas have been shown to exhibit the same cross-sectional imaging characteristics [4]. MRI is the imaging modality for suspected epidural lesions generally and can suggest the presence of infantile hemangioma. They generally demonstrate high signal on T2-weighted images, low signal on T1-weighted images, flow voids suggesting a proliferative phase and intense enhancement post gadolinium. Well-circumscribed tumors and a dumbbell shape can be observed on MR images but are also commonly found with other benign tumors, such as neuromas and meningiomas [21]. Neuraxial hemangiomas rarely demonstrate invasion of the CNS parenchymal [22].

On CT, epidural hemangiomas generally demonstrate hyperdense parenchyma [21, 22].

The imaging appearance of the hemangioma in our case shared many similarities with those of the cases that have been described in the literature. In our case, the mass extended across 8 vertebral segments with symmetrical bilateral foraminal remodeling and protrusion (double sign) and extends along the left iliopsoas muscle. It showed prominent vascularity as shown by the large flow voids on MR images and extensive flow on doppler images. Bone remodeling was seen on CT with the lesion showing high attenuation. The ultrasound characteristics of epidural hemangiomas are unknown; to our knowledge, this is the first case to present the imaging findings of epidural hemangioma on ultrasound. Our case demonstrated an iso to hypoechogenic structure with extensive vascularity on doper images indicating proliferative phase. Follow up MRI in 6 month showed significant decrease in size of the lesion with resolution of the flow voids indicating involuting phase.

It is important to note that imaging has been recommended for hemangioma with atypical presentation such as thrombocytopenia, whenever a cutaneous hemangioma lies over the lumbosacral spine, in perineal hemangiomas with associated urogenital or anal abnormalities, and when more than 4 cutaneous hemangiomas are present [23]. Also, underlying ventral-caudal structural anomalies has been associated with the reticular variant of hemangioma [24].

### Histology

Histologically infantile hemangioma appears as nearly solid masses of small capillaries, consisting of plumps of endothelial cells grouped in well-defined lobules separated by fine strand of connective tissue or by normal intervening tissue sometimes with a feeding artery or neural pseudo-invasion. The lobules are not encapsulated or fibrotic with rare hemosiderin deposition or thrombosis that can sometimes be seen in focal areas of inflammation. This contrasts with congenital hemangiomas that generally have a dense fibrotic stroma, presence of thrombosis or hemosiderin deposition with absent intermingling neovascular, central artery or normal tissue. Importantly, infantile hemangioma express GLUT 1 and LeY on immunostaining, which are not expressed in congenital hemangiomas [11]. The histological distinctions suggest possible distinction on imaging, with the absence of hemosiderin deposition and presence of central flow voids favoring infantile hemangioma over congenital hemangioma.

### Differential

The differential diagnosis of vascular lesions involving the neuraxis in infancy includes hemangioma, hemangioblastoma, vascular malformation, and other neonatal tumors, such as soft-tissue sarcoma [4].

CNS infantile hemangiomas must be distinguished from hemangioblastomas. Hemangioblastomas contain numerous capillaries but also have a rich reticulin network and additional stromal cells that are reactive for S100 and neuron-specific enolase. Moreover, one third of the nervous system hemangioblastomas are associated with VHL syndrome which may add in the clinical distinction between the two [21, 25, 26].

AVMs demonstrate lack of soft-tissue mass, prominent flow voids, growth proportionate with the child’s age, and early venous shunting. Unlike hemangioma, soft-tissue sarcoma is often heterogeneous and exhibits invasive characteristics. Other neonatal tumors such as neuroblastoma, plexiform neurofibroma, and ganglioneuroma do not regress in the manner of infantile hemangioma [4].

Moreover, many published case reports and series discuss “capillary hemangiomas” and “cavernous hemangiomas” many of which are described in older pediatric patients [21, 27–35]. Unfortunately, the literature here dose not adhere strictly to the ISSVA classification with the lesions mostly not exhibiting the classical proliferative and involuting phases of infantile hemangiomas with no routine testing for the GLUT1 expression. This makes a precise categorization of the nature of these vascular lesions problematic.

That being said, spinal epidural hemangiomas could be considered as part of the differential diagnosis for a dumbbell-shaped foraminal mass, along with schwannomas, meningioma [36, 37]. The latter two are especially important in older pediatric patients and adults. Epidural hemangiomas generally exhibit homogenous and intense contrast enhancement, whereas schwannomas can demonstrate cystic component and tend to show less enhancement. On T2-weighted sequence, epidural hemangiomas appear hyperintense, whereas meningiomas are typically isointense. For dumbbell-shaped lesions with enlargement of the neural foramen and radicular pain, schwannoma is more come than an epidural hemangioma in older patients [38].

### Treatment

In the literature, surgical excision seems to be the treatment of choice when the diagnosis is referred to as “epidural hemangioma” with gross total resection considered the “goal” with “typical good prognoses.” This is especially true when the term is used in the older pediatric and adult population [28, 39–44]. Only one previously published report of a preoperative embolization was identified, without significant intraoperative benefit [41, 45]. Moreover, the muddled terminology might be conflating distinct entities leading to miss characterization and miss treatment.

However, in neonates and infants the behavior of infantile hemangioma within the CNS space should theoretically mimic the lesion’s behavior outside, both in its natural history and response to therapy. This is supported by multiple studies showing good response to treatment with medical therapy. Ezra A. Burch (2015) presented a case of a child with infantile hemangioma on the back, who had invasion of the spinal column shows synchronous regression of both the cutaneous and extra-cutaneous infantile hemangioma after corticosteroids treatment [5]. Another case report to the same effect showed involution of cutaneous and intraspinal infantile hemangioma in response to the beta-blocker propranolol [46]. Viswanathan et al. (2009) also showed regression of the prespinal and intraspinal extrathecal extension of an epidural hemangioma in response to corticosteroid treatment [4]. Similar results were shown by Hernandez-Martin et al. (2008) [47]. Hervey-Jumper et al. (2011) described a newborn treated with partial resection of a L5-S1 hemangioma, with follow-up images showing gradual resolution of the residual component with complete resolution after 16 month without therapy [48]. Yu et al. (2016) document successful treatment with beta-blockers [49].

Medical therapy might not be always of value, for example V. Viswanathan et al. (2009) present a 10-month-old male infant with lumber hemangioma had no change in the hemangioma after corticosteroid therapy. However, another case of a 4-month-old female also presented by V. Viswanathan (2009) showed decrease in size after corticosteroids [4].

Our cases showed near complete response at the time of follow up highlighting, as for similar cases, the value of medical treatment in neonatal patients and infants.

## Conclusion

Infantile hemangiomas are endothelial tumors rarely involving the spine. The classification and nomenclature used to describe vascular lesions has been a source of confusion with interchangeable use of various terminology and variability between the radiologist, pathologist, and neurosurgeon. Due to the clinical implications and different aggressiveness of the treatment options, proper diagnosis of intraspinal/epidural infantile hemangioma is key. Clinical history of a neonatal vascular lesion with a proliferative and involuting phase, supported by radiological features of enhancing intra-spinal mass with flow voids, sometimes in association with an extra-spinal component, should hint toward the diagnosis of an infantile hemangioma. Histological evaluation with GLUT 1 and LeY immunostaining is strongly recommended for the purpose of reaching a conclusive diagnosis, since this has a drastic effect on the management, perhaps making beta blockers, a very accessible medication, a first line therapy. Finally, both surgical and medical treatments should be considered with further research on the subject is needed.
